# Prostate cancer

**DOI:** 10.1038/sj.bjc.6600962

**Published:** 2003-05-27

**Authors:** John Masters

**Affiliations:** 1 University College London, UK

Is there someone whom you want to give a generous (about £100) Christmas or birthday present to who treats prostate cancer? A urological surgeon or oncologist with a particular interest in the management of this disease will welcome this book as an addition to their coffee table or office.

‘Prostate Cancer’ is a compilation of chapters, mainly dealing with the clinical aspects of management, with a few chapters touching on the biology of the disease. There is even a short chapter on the palliative care of men with prostate cancer, currently the inevitable outcome for 3–4% of the male readers of this review. It must be the first Californian textbook of prostate cancer, written almost exclusively by people working now or recently in California, and at times this restriction gives the book a parochial outlook. Indeed, there are some interesting words of warning for Asian men who seek their fortune in Los Angeles (LA) – Japanese men living in LA increase their risk of being diagnosed with prostate cancer five-fold and Chinese men 10-fold. Of course, many of the top names in prostate cancer do live and work in California and have contributed chapters. But there are perhaps one or two people outside this state who understand aspects of prostate cancer management, and these experts could have made the book even more enjoyable and instructive to read.

There seems to have been a flood of books on prostate cancer, and the clinical scientist with a general interest is spoilt for choice. Indeed, the first editor of this volume is competing with himself for much the same market in ‘Prostate Cancer: Principles and Practice’, published by Lippincott, also in 2002, which really is a comprehensive treatise on the subject.

Some of the chapters are gems in themselves. The imaging chapter is particularly instructive and well-illustrated, and the contribution on model systems by Yuzhuo Wang and two of the big guns in this field, Gerald Cunha and Simon Hayward, must be worth getting hold of even if you are not interested in the rest of the chapters. Those readers who failed to achieve their ambition to be surgeons might be tempted to follow the step-by-step pictures to have a go at radical prostatectomy.

The editors appear to have left the individual texts fairly untouched. For example, it is said that nearly 200 000 men develop prostate cancer in the US each year and over 30 000 die of it, and this is the gist of the first or second paragraph of most of the chapters. Various typographical errors escaped the red pen, although these are not sufficiently frequent to spoil the read. Perhaps the biggest problem with collections of chapters such as these is that the most efficient authors get the poorest deal, as their chapters are often out of date before the book appears. For instance, there is not one reference from this century in the chapter on molecular biology.

There is some other repetition, particularly in relation to prostate specific antigen (PSA), but this overlap is inevitable and allows each chapter to be read in isolation. It is interesting that 50% of men with organ-confined cancers (ie men who are potentially cured by surgery or radiotherapy) can present with serum PSA values below the normal level (4 ng ml^−1^). Also, prostate cancer is found in only about one-third of men with serum PSA values above 4 ng ml^−1^, but below 10 ng ml^−1^ (the upper limit for some surgeons to offer radical prostatectomy). These figures illustrate what a poor diagnostic test the serum PSA value is for prostate cancer, in terms of both specificity and sensitivity.

This book is a beautifully presented glossy volume, part of the American Cancer Society Atlas of Clinical Oncology series, with stunning intraoperative colour photographs. Your clinical colleague will be delighted to receive a copy, and will also have the benefit of an attached CD that they can download onto their computer.

